# Strong evidence for the evolution of decreasing compositional heterogeneity in SARS-CoV-2 genomes during the pandemic

**DOI:** 10.1038/s41598-025-95893-z

**Published:** 2025-04-10

**Authors:** José L. Oliver, Pedro Bernaola-Galván, Pedro Carpena, Francisco Perfectti, Cristina Gómez-Martín, Silvia Castiglione, Pasquale Raia, Miguel Verdú, Andrés Moya

**Affiliations:** 1 Department of Genetics, Faculty of Sciences, University of Granada, 18071 Granada, Spain; 2 Laboratory of Bioinformatics, Institute of Biotechnology, Center of Biomedical Research, 18100 Granada, Spain; 3 Department of Applied Physics II and Institute Carlos I for Theoretical and Computational Physics, University of Málaga, Málaga, 29071 Spain; 4 Research Unit Modeling Nature, Universidad de Granada, Granada, 18071 Spain; 5 Department of Pathology, Amsterdam UMC, Vrije Universiteit Amsterdam, Cancer Center Amsterdam, Amsterdam, Netherlands; 6 Dipartimento di Scienze della Terra, dell’Ambiente e delle Risorse, Università di Napoli Federico II, Napoli, 80126 Italy; 7 Centro de Investigaciones sobre Desertificación, Consejo Superior de Investigaciones Científicas (CSIC), University of València and Generalitat Valenciana, 46113 Valencia, Spain; 8 Institute of Integrative Systems Biology (I2sysbio), University of València and Consejo Superior de Investigaciones Científicas (CSIC), 46980 Valencia, Spain; 9 Foundation for the Promotion of Sanitary and Biomedical Research of Valencian Community (FISABIO), 46020 Valencia, Spain; 10 CIBER in Epidemiology and Public Health, Madrid, 28029 Spain

**Keywords:** Computational biology and bioinformatics, Evolution, Genetics, Microbiology

## Abstract

The rapid evolution of SARS-CoV-2 during the pandemic was characterized by the fixation of a plethora of mutations, many of which enable the virus to evade host resistance, likely altering the virus’ genome compositional structure (i.e., the arrangement of compositional domains of varying lengths and nucleotide frequencies within the genome). To explore this hypothesis, we summarize the evolutionary effects of these mutations by computing the Sequence Compositional Complexity (SCC) in random stratified datasets of fully sequenced genomes. Phylogenetic ridge regression of SCC against time reveals a striking downward evolutionary trend, suggesting the ongoing adaptation of the virus’s genome structure to the human host. Other genomic features, such as strand asymmetry, the effective number of K-mers, and the depletion of CpG dinucleotides, each linked to the virus’s adaptation to its human host, also exhibit decreasing phylogenetic trends throughout the pandemic, along with strong phylogenetic correlations to SCC. We hypothesize that viral CpG depletion (throughout C➔U changes), promoted by directional mutational pressures exerted on the genome by the host antiviral defense systems, may play a key role in the decrease of SARS-CoV-2 genome compositional heterogeneity, with specific adaptation to the human host occurring as a form of genetic mimicry. Overall, our findings suggest a decelerating evolution of reduced compositional complexity in SCC, whereas the number of K-mers and the depletion of CpG dinucleotides are still increasing. These results indicate a genome-wide evolutionary trend toward a more symmetric and homogeneous genome compositional structure in SARS-CoV-2, which is partly still ongoing.

**Supplementary Information:**

The online version contains supplementary material available at 10.1038/s41598-025-95893-z.

## Introduction

Nucleotide frequencies usually vary along the nucleotide chain, resulting in intragenomic biases^1^. These biases ultimately contribute to the formation of a genome’s compositional structure, which was first uncovered by analytical ultracentrifugation of bulk DNA^2^, as well as through statisticalphysics methods directly analyzing long-range correlations in nucleotide sequences (power spectra, fluctuation analysis in DNA walks and entropic sequence segmentation)^3–5^. The evolution of genome compositional structure has garnered renewed attention in recent years from both theoretical and applied grounds: (1) adequate modelling of compositional heterogeneity is essential for obtaining reliable phylogenetic trees, especially when different lineages exhibit varying nucleotide or amino acid compositions^6^; (2) considering sequence compositional structure has proven to be highly useful in predicting the emergence of SARS-CoV-2 Variants of Concerns (VOCs) with enhanced transmission^7^; and (3) the analysis of genome compositional structure in Cyanobacteria has, for the first time, enabled the discovery of phylogenetic trends driven by natural selection^8^.

Compositional heterogeneities range in size from a few nucleotides to tens of millions of them (see references^9,10^for recent reviews). Arrays of compositional domains of different GC content along the genome sequence form compositional genome structure may be associated with important biological features, such as gene and repeat densities, timing of gene expression, or recombination frequency^2,10^. Genome structure can be changed by any mutational event: point mutations, genome rearrangements, or recombination events. Any population with such a large population size, short generation time, and new environment would likely also accumulate all these changes rapidly, even with a reduced mutation rate. This seems to have occurred in SARS-CoV-2, where, despite its proofreading mechanism and the brief time lapse since its appearance, all these changes have been reported; see ref^11^for recent reviews. Online tracking of SARS-CoV-2 variants and mutations of interest is available on the CoVariants site^7^ (https://covariants.org/).

Mutational events affecting the structural, compositional heterogeneity of a genome can be effectively summarized and quantified by SCC^12^. To achieve this, we first segmented the nucleotide RNA sequence into compositionally homogeneous domains under strict statistical criteria, then accounting for the length and compositional nucleotide differences among the resulting domains by computing its SCC. This measure has been recently employed to determine genome complexity in an ancient and diverse group of organisms, the phylum Cyanobacteria^8^, providing the first evidence for driven evolution towards increasing complexity of genome compositional structure. Tracking changes in the sequence compositional structure of SARS-CoV-2 genomes over time may be relevant on evolutionary and epidemiological grounds. Specifically, the existence of evolutionary trends in the sequence compositional structure of SARS-CoV-2 genomes could reveal whether natural selection is providing adaptation of the virus’s genome structure to the human host.

In this paper, we computed SCC in stratified random datasets of high-quality, wholly sequenced SARS-CoV-2 genomes free of ambiguity symbols (as N, R, Y, S, W). Then, we applied phylogenetic ridge regression to test temporal trends in SARS-CoV-2 SCC evolution^13^. This method has proven effective in revealing both morphological^14^and genomic evolutionary trends^8,15^ trends. We present consistent evidence for a decreasing trend in SCC, indicating a robust long-term adaptive tendency in SARS-CoV-2 evolution. To confirm this notion, we sought links between changes in genome compositional structure and other biological features potentially linked to the virus’s adaptation to its human hosts, such as strand asymmetry, the effective number of *K*-mers, and CpG depletion, which might support the notion that SARS-CoV-2 genomes are evolving to become more symmetric and homogeneous.

## Results

### Compositional genome structure of the SARS-CoV-2

The presence of a compositional structure in the SARS-CoV-2 was first suggested based on detrended fluctuation analyses^16^. Here, using entropic compositional segmentation^17,18^, we found that the SARSCoV-2 genome effectively consists of an array of statistically homogeneous compositional domains with varying lengths and nucleotide frequencies. In particular, the reference genome sequence (hCoV-19/Wuhan/WIV04/2019|EPI_ISL_402124|2019-12-30) consists of seven compositional domains, resulting in a SCC value of 5.7 × 10E^−3^ bits by sequence position. From then on, descendent isolates presented substantial variation in each domain’s number, length, and nucleotide composition. In the stratified dataset of 1063 completely sequenced SARS-CoV-2 genomes analyzed here, the number of segments ranges between 6 and 10 (Supplementary Table 1). Note that genomes with seven segments are the most frequent, while those with 6 or 10 segments occur at lower frequencies. On the other hand, SCC ranges between 4.9 × 10^−3^ and 8.5 × 10^−3^ bits per sequence position on average. Therefore, SARS-CoV-2 genomes show sufficient compositional variation, as detected by SCC, to infer their genealogical or evolutionary relationships.

The strain name, collection date, SCCs, number of segments, asymmetry indexes, CpG and UUG frequencies, as well as other measures for each analyzed genome in the stratified dataset, are shown in Supplementary Table 2. Note that the sample includes SARS-CoV-2 VOCs (Alpha, Delta, and Omicron), minor Variants (Beta, Gamma, Kappa, Iota), as well as no-Variant clades. The density of SARS-CoV-2 VOCs and other clades has changed sequentially throughout the pandemic, with Alpha first appearing in 2020, Delta in 2021, and Omicron dominating from 2022 onward (Supplementary Fig. 1).

A stacked graphical visualization map of the array of segments obtained from each genome is shown in Supplementary Fig. 2. The compositional landscape of the SARS-CoV-2 genome is dominated by six long segments, with shorter, less visible segments scattered along the sequence. A zoomed-in section of the stacked map highlights the variation in segment boundaries across different genomes. Also, note the accumulation of GC-rich segments in the 5’ and 3’ regions of the genome sequence.

## Phylogenetic evolutionary trends

We began investigating evolutionary trends of SCC in SARS-CoV-2 early in the pandemic (April 2020). In the first samples retrieved from the Global Initiative on Sharing All Influenza Data (GISAID)^19–21^, we found no statistical support for phylogenetic trends. However, with the emergence of the first Variants in December 2020, the phylogenetic ridge regression slope of SCC vs. time started to decrease significantly. However, many of those early GISAID entries have ambiguous symbols (mainly N, R, Y, S, and W), which complicate downstream analyses, such as the compositional segmentation of a sequence. To overcome this challenge, we have now chosen to exclusively analyze fully sequenced genomes (i.e., those free of ambiguity symbols). A list of these genome sequences retrieved from the GISAID/Audacity database^19,20^ was compiled as GISAID EPI_SET_240824vr being available at 10.55876/gis8.240824vr. This link allows us to recover not only the RNA sequences but also all metadata associated with them. Here, we present results from a stratified random subsample of 1063 completely sequenced genomes from around the globe^19,20^ The obtained SCC values, number of segments, collection dates, accession numbers (EPI_ISLs), and other relevant data are shown in Supplementary Table 2.

To infer the phylogeny, SARS-CoV-2 genome sequences were aligned using *MAFFT*^22^ (with the options *thread − 1* and *nomemsave*). The best ML tree was inferred using *IQ-TREE 2*^23^using the GTR nucleotide substitution model^24,25^ (with the options *GTR + F + R2*). To solve polytomies, we used the function *fix.poly* from the *RRphylo*package^13,26,27^. The least-square dating (LSD2) method^28^ was used to build a time-scaled tree. Finally, we rooted the inferred time tree to the GISAID SARS-CoV-2 reference genome (hCoV-19/Wuhan/WIV04/2019|EPI_ISL_402124|2019-12-30).

**Fig. 1:**
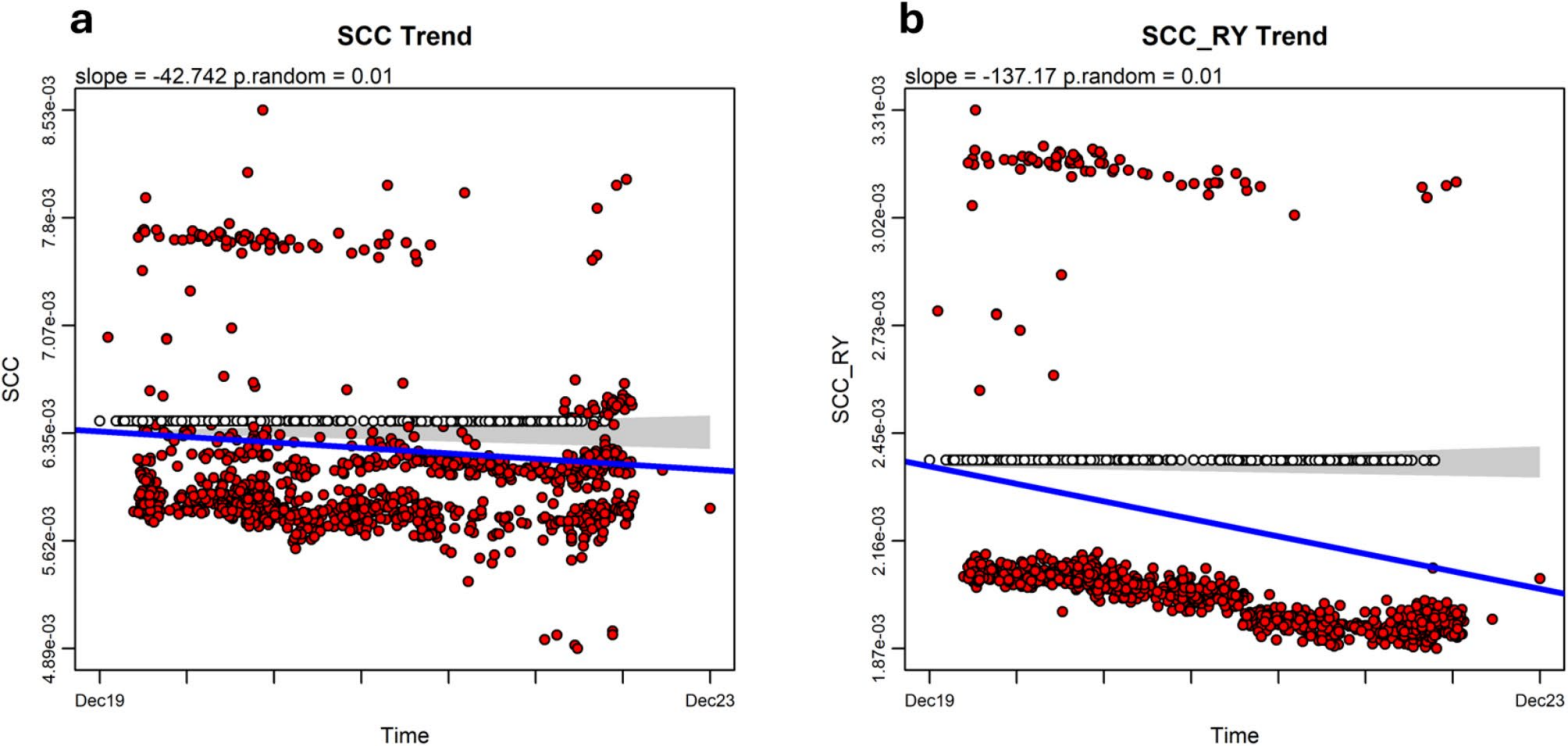
Phylogenetic regressions of SCC (**a**) and the partial complexity SCC_RY (**b**) against pandemic time (days since the first SARS-CoV-2 was isolated), as determined by the RRphylo package^13^. The regression slopes are both significantly lower than with BM expectation (*p* < 0.01), indicating a decreasing trend over time for SCC and SCC_RY. Red dots are tip values; open circles are fitted ancestral states. The grey area is the 95% confidence interval of the distribution of BM slopes, and the blue line is the regression slope.

To test for evolutionary trends in compositional complexity, we used the function *search.trend*^29^in the RRphylo R package^13^. The function computes the regression between compositional complexity and time since the virus tree root. It contrasts the realized slope of this regression against a family of 1,000 slopes generated under the Brownian motion (BM) model, which models evolution as if there were no trend and a single evolutionary rate constant across the tree. The regression slope of SCC versus time is significantly lower than with BM expectation (*p* < 0.01), indicating a decreasing trend over time (Fig. 1a; Table 1). We further tested this notion by applying a Brownian Motion with Trend (BMT) test using the package geiger^27^. BMT is a modified Brownian motion (BM) model whereby the trait mean is allowed to drift over time. We found circumstantial confirmation for the decreasing trend indicated by *search.trend*. BMT suggests a negative trend (decrease over time) and has lower AIC than ordinary BM (AIC_BMT_ = −12998.195, slope = −0.316; AIC_BM_ = −12997.511), but the difference with BM is not statistically significant (likelihood ratio test, LRT p-value = 0.10, Table 1).). We have also investigated the phylogenetic trend of the partial complexity SCC_RY (Fig. 1b), which is one of the partial complexities in which SCC can be decomposed^30^. The behavior of SCC_RY is potentially attractive because it mainly reflects strand asymmetries in the distribution of purine/pyrimidines along the genome sequence, which have been related to key biological mechanisms, including protein binding preferences, transcription factor interactions, retrotransposition, DNA damage and repair preferences, transcription-replication collisions, and mutagenesis mechanisms^31^.

By using *search.trend*, we found that SCC_RY regression slope is significantly lower than with BM expectation (*p* < 0.01), indicating a decreasing trend over time as with for SCC (Fig. 1b; Table 1). BMT test, compared to BM, confirms this notion (AIC_BMT_ = −14178.889, slope = −0.212; AIC_BM_ = −14177.235, LRT p-value = 0.05, Table 1).

**Table 1:** Results of the evolutionary model fitted for SCC, SCC_RY, SCC_SW, SCC_KM, PR_K1, S1_K1, CpG, UUG. Each metric was regressed against time with RRphylo function *search.trend* to test for Temporal trends in the metric values, either computing the regression of the metric against time as is (p.real) and ranking the real data slope against a family of 100 randomly generated slopes obtained simulating the brownian motion model of evolution. In addition, we fitted the brownian motion model of evolution by either ignoring (BM) or admitting (BMT) the existence of a trend term ‘trend’ in the metric over time. BM is compared to BMT by means of a likelihood ratio test, whose P.value is reported in the table (LRT_p). The phylogenetic signal and its significance are calculated to fit Blomberg’s K value (P indicates the P.value that K differs from 0, i.e., there is no phylogenetic signal in the data).

	search.trend			Brownian motion with and without trend				trend suggested by both search.trend and BMT	phylogenetic signal	
GENOME TRAIT	slope	*p*.real	*p*.random	AIC.BM	AIC.BMT	trend	LRT_p		K	*P*
SCC	−42.74	0.00	0.01	−12997.511	−12998.195	−0.3	0.101	decrease	0.283	0.078
SCC_RY	−137.17	0.00	0.01	−14177.235	−14178.889	−0.2	0.056	decrease	0.442	0.024
SCC_SW	3.92	0.38	0.63	−14772.989	−14774.853	−0.2	0.049		0.258	0.073
SCC_KM	−53.39	0.00	0.01	−14006.345	−14005.913	−0.2	0.209	decrease	0.442	0.022
PR_K1	−187.26	0.00	0.01	−11850.513	−11890.922	−2.1	<< 0.001	decrease	2.387	0.001
S1_K1	−149.19	0.00	0.01	−13319.047	−13345.449	−0.9	<< 0.001	decrease	1.764	0.001
CpG	−27.76	0.00	0.01	6014.193	6016.165	−100	0.844	decrease	0.291	0.022
UUG	23.64	0.00	0.01	4929.028	4930.562	100	0.490		0.266	0.053

The evolutionary rates of SCC and SCC_RY are shown in Supplementary Fig. 3. Both rates increased over time (SCC slope = 5.80 *p* = 0.94; SCC_RY slope = 552.85 *p* < 0.001, Table 2). Compared to randomly generated slopes under Brownian motion evolution, we found that SCC slope is shallower than expected (*p* = 0.01). In contrast, the opposite is true of SCC_RY slope (*p* = 1, notice that in *search.trend*, the p-value is obtained by ranking the actual slope to BM generated slopes so that at *p* > 0.975, the insight is that the real slope is higher than BM expectations, the converse at *p* < 0.025). We further fitted a BMT test where the trend is depicted to occur in rates, rather than in the phenotype, using geiger. We found evidence for decreasing rates for both (SCC: slope in rate regression =−545.24, AIC_BMT_ = −13101.695, LRT p-value < 0.001; SCC_RY: slope in rate regression =−684.446, AIC_BMT_ = −14470.451, LRT p-value < 0.001). However, it must be noted that both SCC (K = 0.28, *p* = 0.078) and SCC_RY (K = 0.44, *p*= 0.022) have a low and marginally significant phylogenetic signal (as measured by Blomberg’s K^32^, Table 2) implying the BM is probably a poor representation of compositional metrics evolution. These results suggest that rates in SCC evolution are probably declining, while the notion for the SCC_RY component is uncertain. Collectively, the analysis of SCC and SCC_RY evolutionary patterns suggests that the virus has gone through an adaptive process to the human host, characterized by weakly declining compositional complexity and decelerating (at least in SCC) evolutionary rates.

**Table 2:** Results of the evolutionary model fitted for the evolutionary rates of SCC, SCC_RY, SCC_SW, SCC_KM, PR_K1, S1_K1, CpG, UUG. Each rate metric was regressed against time with RRphylo function *search.trend* to test for Temporal trends in the metric values, either computing the rate regression of the metric against time as is (p.real) and ranking the real data slope against a family of 100 randomly generated slopes obtained simulating the brownian motion model of evolution. In addition, we fitted the brownian motion model of evolution by either ignoring (BM) or admitting (BMT) the existence of a trend (term ‘trend’) in the rate metric over time. BM is compared to BMT by means of a likelihood ratio test, whose P.value is reported in the table (LRT_p).

	search.trend			Brownian motion with and without trend				trend suggested by both search.trend and BMT
GENOME TRAIT	slope	*p*.real	*p*.random	AIC.BM	AIC.BMT	trend	LRT_p	
SCC	5.81	0.94	0.01	−12997.511	−13101.695	−545.243	<< 0.001	decrease
SCC_RY	552.85	0.00	1.00	−14177.235	−14470.451	−684.446	<< 0.001	
SCC_SW	−530.06	0.00	0.01	−14772.989	−14790.022	−317.441	<< 0.001	decrease
SCC_KM	185.70	0.01	0.27	−14006.345	−14128.078	−561.388	<< 0.001	
PR_K1	2153.57	0.00	1.00	−11850.513	−11933.035	1000	<< 0.001	increase
S1_K1	1056.07	0.00	1.00	−13319.047	−13423.167	−548.197	<< 0.001	
CpG	653.81	0.00	1.00	6014.193	5869.966	1000	<< 0.001	increase
UUG	354.39	0.00	1.00	4929.028	4891.79	−407.604	<< 0.001	

## Biases in K-mer distribution

To gain insight into the biological significance of the observed compositional evolutionary trends, we further investigated other genomic features that follow similar temporal dynamics. The first of these features is the bias in the distribution of *K*-mers, which are substrings of length *K* that serve as fundamental units for analyzing and comparing genomic sequences. The distribution of *K*-mers within a genome sequence holds significant biological relevance, as it provides insights into genomic compositional structure and function^33,34^.

## Strand asymmetry

**Fig. 2:**
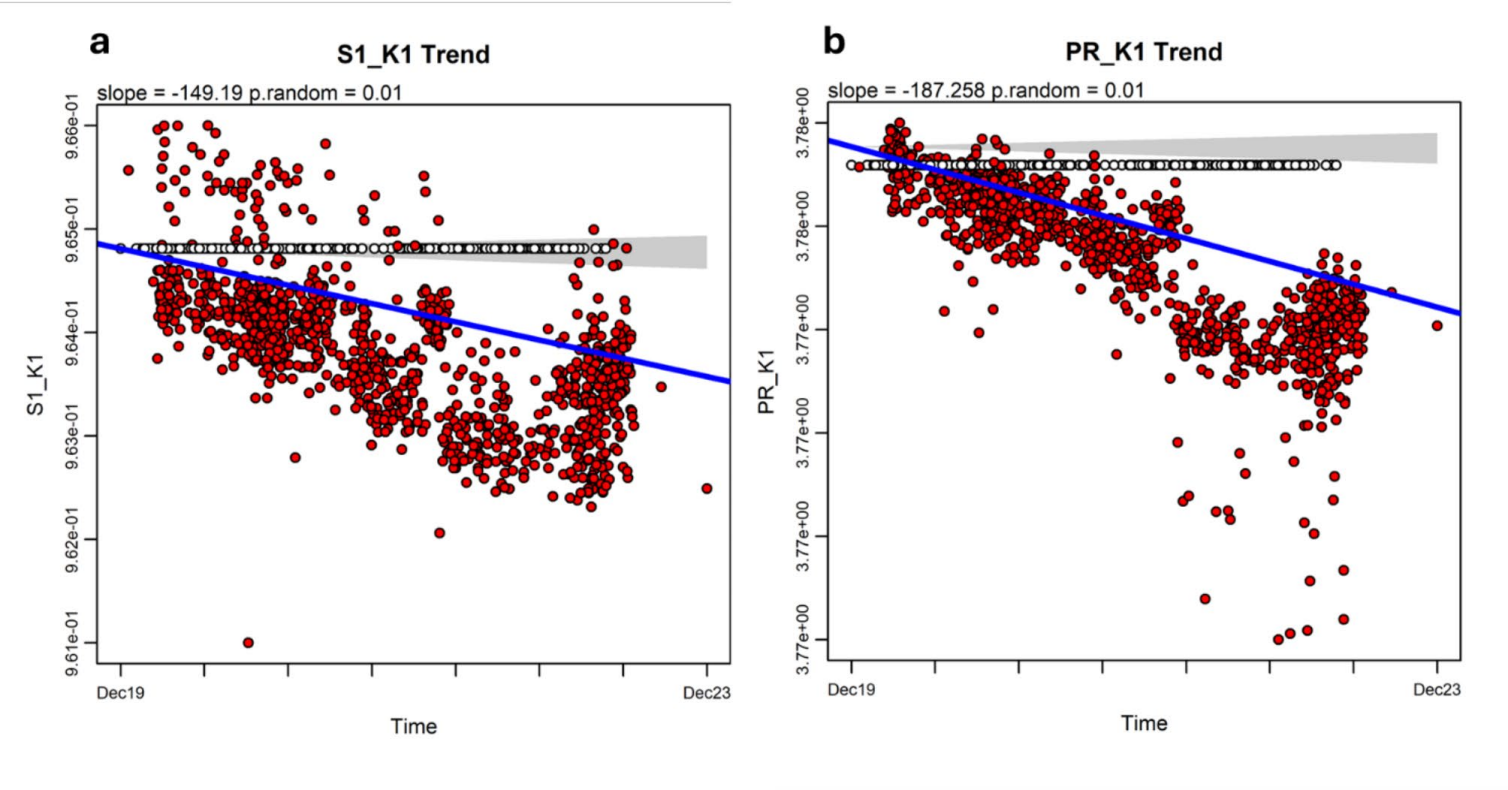
Phylogenetic regressions for *K*-mer (*K*= 1) distribution, as measured by the S^1^strand asymmetry index (a) and the Participation Ratio (PR (b), reveal strong phylogenetic decreasing trends over time. The RRphylo package^13^ was used. See the caption of Fig. 1 for annotations.

Seeking out for biases in the distribution of *K*-mers, we first used the S^1^asymmetry index^35^ for *K*= 1 to 6. Using phylogenetic ridge regression of S^1^against time, we observed a highly significant decreasing trend in S^1^ for *K* = 1 (Fig. 2a, slope = −149.18, *p* < 0.001, Table 1) and, to a lesser extent, for *K* = 3 (not shown). BMT test holds the same insight (AIC_BMT_ = −13345.449, slope = −0.88; AIC_BM_= −13319.047, LRT p-value < 0.001), strengthening the notion that the asymmetry index strongly decreases over time. The phylogenetic signal for S^1^ is high and significant (K = 1.764, *p* < 0.001, Table 1). Analysis of S^1^ rate values points to the existence of a strong trend for increased rates, yet it is positive using *search.trend* (slope = 1056.07, *p* = 1), but negative under the Brownian motion model (AIC_BMT_ = −13423.167, slope = −548.20, LRT p-value < 0.001, Table 2).

## The participation ratio

A second bias in the distribution of K-mers that we tested was the effective number of K-mers, represented as the Participation Ratio (PR). We observed highly significant decreasing trends for all *K* values, save *K* = 2. The steepest negative slope was observed for *K* = 1 (Fig. 2b). With such a K value, *search.trend* regression of PR against time gives slope = −187.258 and p-value = 0.01. BMT test provides further support (AIC_BMT_ = −11890.922, slope = −2.14; AIC_BM_ = −11850.513, LRT p_−_value < 0.001, Table 1). The regression results of rates of PR evolution against time point to a strong trend for increasing rates over time (*search.trend* slope = 2153.57, *p* = 1; AIC_BMT_ = −11933.035, slope = 1000, LRT p-value < 0.001, Table 2).

These results strongly support the notion that the number of *K*mers effectively used by SARS-CoV-2 decreased during the pandemic at progressively faster rates, providing a continuous simplification and homogenization of the virus genome.

### CpG depletion

**Fig. 3:**
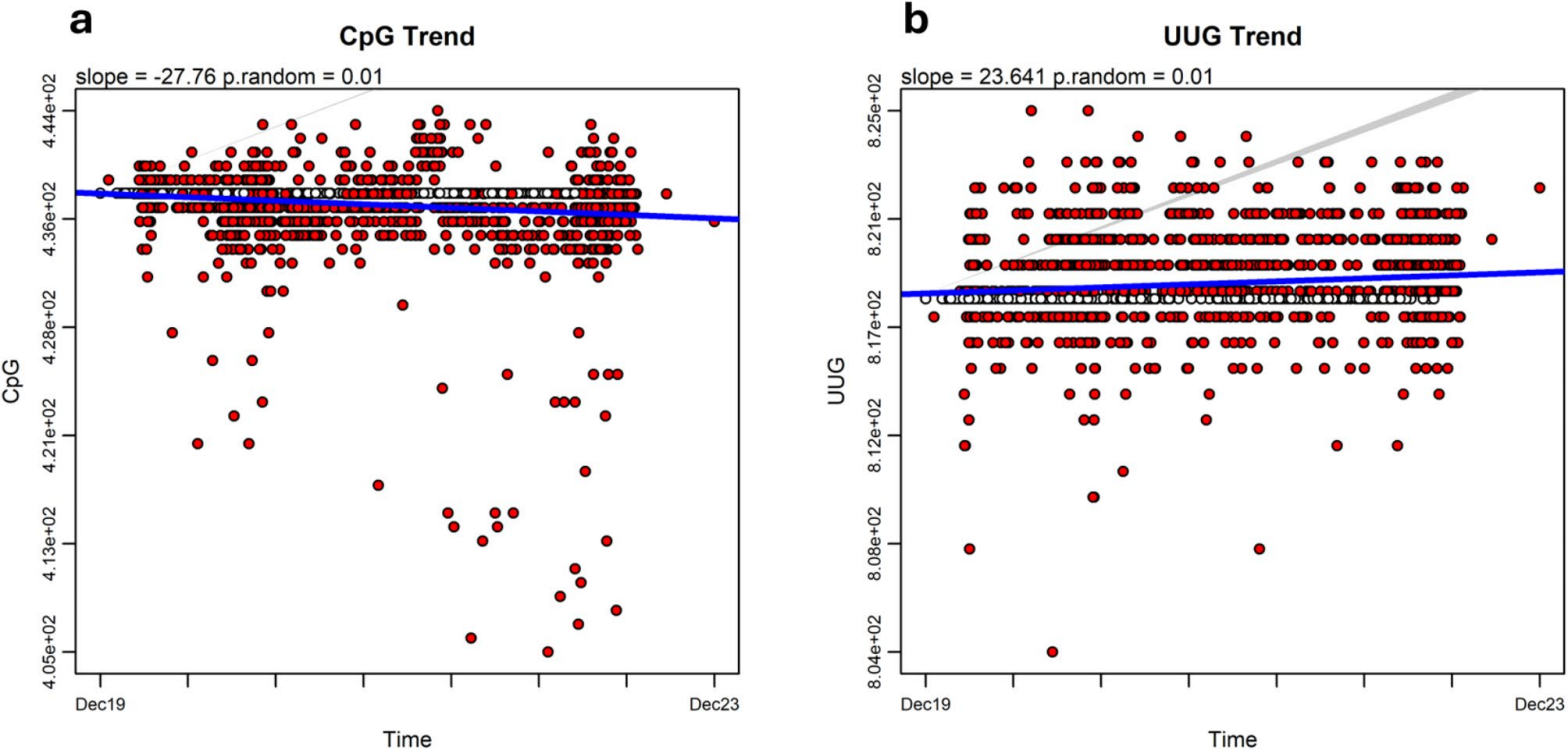
A modest but significant decreasing phylogenetic trend in CpG frequencies (a) and a highly significant increasing trend in the frequencies of their deamination product (UUG) (b) were observed in SARS-CoV-2 genomes. The RRphylo package^13^ was used. See the caption of Fig. 1 for annotations.

Single-stranded RNA viruses replicating in vertebrate hosts tend to have a low frequency of CpG dinucleotides in their genomes^36^. Moreover, in SARS-CoV-2, a gradual decline in CpG content has been observed^37^, albeit at a modest rate over time. Interestingly, we applied phylogenetic regression to CpG frequencies in our SARS-CoV-2 dataset and found a weak but significant decreasing trend (*search.trend*: slope = −27.76, *p* = 0.01, Fig. 3a; Table 1), which is still confirmed by BMT (slope = −99.7, AIC_BMT_ = 6016.165) This model, though, is not statistically superior to a simple BM (AIC_BM_ = 6014.193, LRT p-value = 0.84). The rate regression results point, as with PR, to strongly increasing rates over time (*search.trend* slope = 653.81, *p* = 1; AIC_BMT_ = 5869.966, slope = 1000, LRT p-value < 0.001, Table 2). For UUG, we found an increasing pattern in *search.trend* (slope = 23.64, *p* = 0.01, Fig. 3b). As with CpG frequencies, BMT test indicates no difference from pure Brownian (no trend) motion prediction (BMT: slope = 100, AIC_BMT_ = 4930.562; BM: AIC_BM_ = 4929.028, LRT p-value = 0.49). The rate regression results point, as with CpG, to strongly increasing rates over time (*search.trend* slope = 354.29, *p* = 1), which, however, is not found under BMT (AIC_BMT_ = 4891.79, slope = −407.6, LRT p-value < 0.001, Table 2).

Two explanations for CpG depletion, based on directional mutational pressures exerted on the SARSCoV-2 genome by host antiviral defense systems, have been proposed^37^. The first attributes CpG depletion to the deamination of methylated cytosines by the host methyltransferases^38^. However, SARS-CoV-2 does not have a DNA stage, meaning this explanation is unlikely Click or tap here to enter text^39^. A second biological mechanism^40,41^ more clearly explaining the decreasing trends in SCC*s*may be the combined actions of APOBEC (apolipoprotein B mRNA editing catalytic polypeptide-like proteins, which are zinc-dependent deaminases^42^) and ZAP (zinc-finger antiviral protein^43^). The catalytic activity of APOBEC enzymes leads to the transformation of 5’-UCG-3’ sites into 5’UUG-3’ via cytosine deamination, effectively removing the ZAP recognition site (5’-CG-3’). This deamination, changing C➔U, enables viral RNA to evade degradation by ZAP. Over time, a decrease in CpG dinucleotides and a corresponding increase in their deamination product (UUG) are expected. The decreasing phylogenetic trend we observed in CpG frequencies (Fig. 3a), coupled with the corresponding increase in UUG trinucleotides (Fig. 3b), aligns well with this mechanism. It must be noted that although neither CpG nor UUG differ significantly from BM expectations, they differ from each other in terms of the sign of the regression slope, meaning their evolution points towards opposite directions over the same period. We verified this statistical fitting by a generalized least squares (GLS) model using the function gls in the package nlme^44,]^ setting the variable as a dummy. The GLS model interaction is highly significant, and the GLS slope *β* has opposite sign as the interaction term *β*_*interaction*_ (*β *= 496.4, *β*_*interaction*_ = − 1579.1, p_interaction_< 0.001). This indicates the C➔U deamination is a feasible mechanism conferring adaptation of the SARS-CoV-2 genome to humans^45^.

### Phylogenetic correlations of SCCs to other biological features

To further investigate the association of SCC*s*to other biological features with similar temporal dynamics, we constructed a Phylogenetic Generalized Least Squares (PGLS) regression model^46^ for each of the four SCC*s*as the dependent variable, and strand asymmetry (S^1^ index, *K* = 1), the effective number of *K*-mers (PR, K = 1) and the frequencies of CpG dinucleotides and UUG as the independent variables. We used the RRphylo function *PGLS_fossil*to perform the regression analysis^47,,48,49^. The aim here is to understand the significance of each independent on the dependent variable while accounting for phylogenetic relationships. All phylogenetic correlations obtained from the PGLS models were highly significant at P < ≪ 0.001, regardless of which aspect of SCC is analyzed, with the sole exception of UGG, which is associated with SCC_KM and marginally to SCC_RY, but not to SCC or SCC_SW (Table 3). Additionally, the results in Table 3 suggest that S^1^*_K1* has the strongest positive effect, while PR_K1 shows a negative impact, both being important predictors of SCC_RY.

**Table 3:** PGLS model results obtained regressing metrics of SCC as the dependent variables against S^1^_K^1^(S^1^, K = 1), PR_K1 (PR, K = 1), CpG, and UGG.

		Estimate	St Error	t.value	*p*.value
SCC	(Intercept)	0.73	0.08	9.19	<< 0.001
	CpG	0.00	0.00	23.55	<< 0.001
	PR_K1	−0.37	0.02	−19.69	<< 0.001
	S1_K1	0.67	0.02	29.75	<< 0.001
	UGG	0.00	0.00	−1.34	0.18
SCC_RY	(Intercept)	0.393	0.037	10.567	<< 0.001
	CpG	0.000	0.000	24.167	<< 0.001
	PR_K1	−0.222	0.009	−25.120	<< 0.001
	S1_K1	0.447	0.011	42.566	<< 0.001
	UGG	0.000	0.000	−1.650	0.099
SCC_SW	(Intercept)	0.28	0.04	6.96	<< 0.001
	CpG	0.00	0.00	22.04	<< 0.001
	PR_K1	−0.13	0.01	−13.75	<< 0.001
	S1_K1	0.21	0.01	18.30	<< 0.001
	UGG	0.00	0.00	−0.59	0.55
SCC_KM	(Intercept)	0.416	0.044	9.456	<< 0.001
	CpG	0.000	0.000	21.716	<< 0.001
	PR_K1	−0.234	0.010	−22.326	<< 0.001
	S1_K1	0.469	0.012	37.706	<< 0.001
	UGG	0.000	0.000	−2.264	0.024

## Discussion

The great number of point mutations, genome rearrangements, and recombination events observed in SARSCoV-2 have resulted in a notable diversification of the virus as it adapted to the human host during the pandemic^50,51^. Many of these changes, particularly those leading to the emergence of VOCs, may be adaptive. Examples include inter-lineage recombinants^52^, mutations enabling VOCs to neutralize host resistance or escape antibodies^53^, consequently increasing transmissibility (a paradigmatic example being the outbreak of the Omicron Variant), co-mutations^54^that become more prevalent worldwide compared to single mutations, primarily responsible for temporal changes in transmissibility and virulence, as well as parallel mutations in multiple independent lineages and Variants^55^, which are of particular interest in the context of adaptation of SARS-CoV-2 to the human host. Structural mutations revealed by homology modeling experiments, which can potentially alter the number or nucleotide frequencies within the array of compositional segments of the SARS-CoV-2 genome, as well as higherfitness mutations, such as those in the *nucleocapsid* or *spike*genes, along with hitchhiking mutations in other genomic regions, may also play a role^56^.

We focus here on the potential effects that all these changes may have had on the evolution of the compositional genome compositional structure of SARS-CoV-2. To this end, we computed SCC^12^and SCC partial complexities^30^, capturing the evolution of the virus’s genome structure in near real-time. Despite its short length (~ 29,900 nt), the SARS-CoV-2 genomes analyzed here are segmented into 6 to 10 compositional domains (~ 0.25 segments by 1000 nt on average; see column *nseg*in Supplementary Table 2). Although such segment density is lower than in free-living organisms (like cyanobacteria, where an average density of 0.47 segments by 1000 nt was observed^8^), the compositional variability we found in the SARS-CoV-2 may be sufficient for comparative evolutionary studies of genome structure, which could shed light on the origin and evolution of the COVID-19 pandemic^56,57^.

Phylogenetic ridge regression of SCC and SCC_RY over time revealed decreasing evolutionary trends in sequence compositional complexity (Fig. 1), along with increasing rates of change (Supplementary Fig. 3), suggesting the ongoing adaptation of virus’s genome structure to the human host. Notably, applying the same method to other genomic features with similar temporal dynamics—such as strand asymmetry (Fig. 2a), the effective number of *K*-mers (Fig. 2b), and CpG depletion (Fig. 3a), all of which are potentially linked to key biological features—also reveals decreasing phylogenetic trends over time. The strength of the relationship between SCCs and these other biological features was checked by PGLS models^46^, where each SCC served as the dependent variable and strand asymmetry, the effective number of *K*-mers, and CpG depletion were the independent variables. AIC criterium indicates that the model fit for SCC_RY (Table 1) provides the best explanation for the variation across the SARS-CoV-2 phylogeny.

The decreasing phylogenetic evolutionary trends observed in SCC, SCC_RY, strand asymmetry, the effective number of *K*mers, and CpG depletion (Figs. 1, 2 and 3) are particularly interesting, as they suggest that the virus’s ongoing adaptation has been accompanied by a significant reduction in global genome compositional complexity within the global SARS-CoV-2 population, which points to a progressive simplification and homogenization of the SARS-CoV-2 genome’s compositional structure over time. Since SCC*s* integrate the complexity of the entire viral genome, the reductions in SCC*s* could suggest that natural selection is favoring more streamlined, less complex SARS-CoV-2 genomes over time.

In this context, we hypothesize that viral CpG depletion (throughout C➔U changes) promoted by directional mutational pressures exerted on the genome by host antiviral defense systems^40,41^may play a key role in the decrease of genome compositional heterogeneity, with adaptation occurring as a form of genetic mimicry^36,58^. This explanation is consistent with the observed decrease in strand asymmetry, which may indicate optimization of replication efficiency across the genome, with selective pressure favoring specific nucleotide compositions to enhance viral fitness^36,58^. In addition, CpG depletion could also explain the observed decreasing trends in the number of *K*-mers participating effectively (PR) in the observed distribution of K-mers in the SARSCoV-2 sequence. Overall, our findings suggest an evolutionary, genome-wide trend toward a more symmetric and homogeneous compositional structure in the SARSCoV-2 genome. This reflects an adaptive process mainly driven by natural selection acting on CpG composition as the virus continues to specialize to the human host. Further experiments are needed to confirm the adaptation process of SARS-CoV-2 as a form of genetic mimicry. For example, one could compare the metrics calculated for SARS-CoV-2 with the metrics one would get for the human genome. Simulation experiments introducing C➔U changes in random sequences and observing how the SCC evolves would also be helpful.

In conclusion, we prove that the increase in fitness of Variant genomes, associated with higher transmissibility, may have contributed to a reduction in SARS-CoV-2 sequence compositional heterogeneity throughout the pandemic. This genome compositional dynamic may have been driven by the rise of highly fit viral variants and convergent evolution, contributing to an adaptive specialization process in the human host through natural selection acting on CpG frequencies as a form of genetic mimicry^36,58^. Adaptation processes have been observed in codon usage and amino acid preferences in other viruses^59^. Further monitoring of these evolutionary trends in current and emerging Variants and recombinant lineages^60,61^, using the methodology applied here, may help clarify whether—and to what extent—the evolution of compositional genome structure in this and other pathogen genomes affects human health.

## Methods

### The genome of SARS-CoV-2

The SARS-CoV-2 genome is an approximately 30 kb, positive sense, 5’ capped single-stranded RNA molecule^62^. An updated genomic map of the isolate Wuhan-Hu-1 (MN908947.3) of SARS-CoV-2 we used as a reference genome for sequence alignment is available at https://www.ncbi.nlm.nih.gov/nuccore/MN908947.3?report=graph. Genomic information on the official reference sequence employed by GISAID (EPI_ISL_402124, hCoV-19/Wuhan/WIV04/2019, (WIV04)) is available at https://www.gisaid.org/resources/hcov-19-reference-sequence/. We used this genome as the root when inferring the SARS-CoV-2 phylogeny. Note that although WIV04 is twelve nucleotides shorter than Wuhan-Hu-1 at the 3’ end, the two sequences are identical in practical terms; the 5’ UTR is the same length, and the coding regions are identical. Therefore, the coordinates and relative changes stay the same whichever sequence is used, which is relevant to extracting the coordinates of compositional segments.

### Retrieving a stratified dataset of SARS-CoV-2 genomes, free of Ns and other ambiguous symbols

SARS-CoV-2 genome sequences are available from the GISAID/Audacity database^19,20^. However, many of them are not fully sequenced and have ambiguous symbols (N, R, Y, S, W, K, M), which could complicate the compositional segmentation of a sequence. To overcome this difficulty, on September 25, 2024 we downloaded the entire global phylogeny for 12,647,126 high-quality sequences as a Newick tree file, along with their associated metadata (metadata.csv), from the GISAID website (https://www.epicov.org/epi3/frontend#e90a5). We then randomly shuffled the rows in metadata.csv and extracted the first 10,000 rows to establish the initial random sample with high-quality genomes from around the globe, then discarding duplicates and entries with incomplete collection dates. In addition, by using *seqtk* (https://github.com/lh3/seqtk) and *Nextclade*^63^ software programs, we further filtered to discard sequences containing Ns and other ambiguities, thus obtaining a filtered sample with 4,336 completely sequenced genomes spanning from December 2019 to January 2024. A list of these 4,336 genome sequences was compiled as GISAID EPI_SET_240824vr, being available at 10.55876/gis8.240824vr.

Preliminary analyses of the above sample reveal phylogenetic trends for SCCs (see the preprint: https://www.biorxiv.org/content/10.1101/2024.12.03.625388v1). However, we recognized that the sample with 4,336 genomes (extracted by uniform random sampling) has a highly disproportionate number of genomes from the USA and England and a disproportionately low number of genomes from less developed but more populous nations with widespread COVID, which may introduce biases into the phylogenetic regression analysis. These biases were partially corrected by using stratified sampling methods (https://www.spsanderson.com/steveondata/posts/2024-07-29/) rather than uniform random sampling. In this way, using a Python script (available at the Zenodo repository), we obtained stratified subsamples with a more balanced number of genomes by country, yet showing similar phylogenetic trends as the original sample. This retrieval workflow was used to get different stratified subsamples, ensuring the repeatability of our analyses. Here, we present the analysis of one of these stratified subsamples, consisting of 1,063 completely sequenced genomes free of ambiguous symbols (Supplementary Table 2).

### Sequence Compositional Complexity (SCC)

The sequence compositional structure of each SARS-CoV-2 genome was determined by computing its Sequence Compositional Complexity (SCC)^12^, which consists of a two-step process: the nucleotide sequence was first segmented into homogeneous, statistically significant compositional domains, followed by the computation of SCC. Using the alphabet {A, T, C, G} (remember that in RNA genomes, the letter T is used to denote Uracil (U)), we divided each SARS-CoV-2 sequence into an array of compositionally homogeneous, non-overlapping domains using a heuristic, iterative segmentation algorithm^17,18^. In brief, a sliding cursor is moved along the sequence, and the position that optimizes a proper measure of compositional divergence between the left and right parts is selected. We choose the Jensen-Shannon divergence (Eqs. [1] and [2] in^17^) as the divergence measure, as it can be directly applied to symbolic nucleotide sequences. If the divergence is statistically significant (at a given significance level that we choose to be *s =* 0.95), the sequence is split into two segments. Note that each pair of resulting segments is more homogeneous than the original sequence. The two new segments are then independently subjected to another round of segmentation. The process continues iteratively over the new segments while sufficient significance continues appearing.

Note that the *s *value (here 0.95) is the probability that the difference between adjacent domains is not due to statistical fluctuations. Recent improvements to the segmentation algorithm^64^ allow segmenting sequences with long-range correlations, as those recently reported in the SARS-CoV-2 ^16^. The result is the segmentation of the original sequence into an array of contiguous, non-overlapping segments (or compositional domains) whose nucleotide composition is entropically homogeneous within a predefined level of statistical significance, *s.* A stacked map of the segmentations observed in all the genomes within the analyzed dataset is presented in Supplementary Fig. 2.

Once a sequence is segmented into an array of homogeneous compositional domains at a given significance level (e.g., p-value ≤ 0.05), a measure of Sequence Compositional Complexity or SCC^12^, expressed in bits by sequence position, was computed:

1 $$\:SCC=H\left(S\right)-\sum\:_{i=1}^{n}\frac{{G}_{i}}{G}H\left({S}_{i}\right)$$

where *S* denotes the whole genome sequence, G is its length, and G_i_ is the length of the *i*^th^ domain *S*_*i*_. 

$\:H\left(\bullet\:\right)=\:-\sum\:f{log}_{2}f$ is the Shannon entropy of the distribution of relative frequencies of symbol occurrences, *f*, in the corresponding (sub)sequence. It should be noted that the above expression is the same as the one used in the segmentation process, applying it to the tentative two new subsequences (*n*= 2) to be obtained in each step. In this way, the segmentation procedure finds the partition of the sequence that maximizes SCC. It is also worth noting that the two steps of the SCC computation are based on the same theoretical background. Note that (1) this measure is zero if no segments are found in the sequence (the sequence is compositionally homogeneous, e.g., a genuinely random sequence) and (2) it increases/decreases with both the number of segments and the degree of compositional differences among them. In this way, the SCC measure is analogous to the method proposed by^65^ for estimating complexity in morphological characters: an organism is more complex if it has a greater number of parts and a higher differentiation among these parts. It is important to emphasize the high sensitivity of this measure to sequence changes. A single nucleotide substitution or one little indel could potentially alter the number, length, or nucleotide frequencies of the compositional domains and, therefore, the resulting value for SCC. A Python script to segment the SARS-CoV-2 genome sequences and compute SCC is available at the repository Zenodo.

### SCC partial complexities

The quaternary alphabet {A, T, C, G} is commonly used for SCC computation. However, taking advantage of the branching property of entropy^66^, SCC can be decomposed into partial complexities by grouping the nucleotides into binary alphabets, as *SW*{GC/AT}, *RY*{AG/TC} or *KM*{AC/TG}^30^. Two of the partial complexities obtained in this way (SCC_SW and SCC_RY) have been directly associated with key biological features. SCC_SW directly reflects changes in GC content, which are often associated with gene and repeat densities, timing of gene expression, or recombination frequency^2,10^ SCC_RY mainly reflects strand asymmetries in the distribution of purine/pyrimidines along the sequence, being related to key biological mechanisms, including protein binding preferences, transcription factor interactions, retrotransposition, DNA damage and repair preferences, transcription-replication collisions, and mutagenesis mechanisms^31^. Nonrelevant biological features have been associated with the alphabet KM {AC/TG}^67^.

### Phylogenetic ridge regression

The phylogenetic ridge regression of SCC was determined by using the *RRphylo*R package^13^. In *Rrphylo*, the change in SCC value between any two consecutive tree branches aligned along a phyletic line is described by the equation *Δ*SCC *= β*_*1*_*1*_*1+*_ *β*_*2*_*1*_*2+*_* .*_*+*_ *β*_*n*_*1*_*n*_ where the *β*_*ith*_ and *l*_*ith*_ elements represent the regression coefficient and branch length, respectively, for each *i*_*th*_ branch along the phyletic line. The matrix solution to find the vector of *β* coefficients for all the branches is given by the equation. $\:\widehat{\beta\:}$ = (**L**^*T*^ **L** + λ**I**)^−1^ **L**^*T*^ SCC; where **L** is the matrix of tip-to-root distances of the tree (the branch lengths), having tips as rows, where entries are zeroes for the branches outside the tip phyletic line, and actual branch lengths for those branches along the path. *λ* is a penalization factor that avoids perfect predictions of SCC, preventing model overfitting. The vector of ancestral states $\:\widehat{a}$ (SCC values at the tree nodes) is obtained by the equation $\:\widehat{a}={\mathbf{L}}^{{\prime\:}}\widehat{\beta\:}$, where $\:{\mathbf{L}}^{{\prime\:}}$ is the node-to-root path matrix, calculated as **L**, but with nodes as rows. The estimated SCC value for each tip or node in the phylogenetic tree is regressed against its age (the phylogenetic time distance, which represents the time distance between the reference genome and the collection date of individual virus isolates) and the regression slope compared to Brownian Motion (*BM*) expectations (which predicts no trend in SCC values and rates over time) by generating 1,000 slopes simulating BM evolution on the phylogenetic tree, using the function *search.trend*^26^ in the *Rrphylo* R package.

### Measuring strand asymmetry (S1)

Strand asymmetry for each SARS-CoV-2 genome sequence was computed using its distribution of *K*mers. One popular method^35^, first computes the *K*th-order strand symmetry of any given sequence as the similarity between its *K*-mer distribution f and the *K*-mer distribution *f’* of its actual or virtual reverse complement. Let us consider the standard four-letter alphabet {A, T, C, G}, then there are 4^*K*^ different *K*-mers. Given the observed distribution of *K*mers in the analyzed sequence, if *f*_i_ stands for the relative frequency of the i-th *K*mer, then $\:{\sum\:}_{i=1}^{{4}^{K}}{f}_{i}=1.\:$In practice, this method used the sum of the absolute values of the differences between *K*mer frequencies:

2 $$\:{S}^{1}=1-\frac{\sum\:_{i}\left|{f}_{i}-{f{\prime\:}}_{i}\right|}{{\sum\:}_{i}\left|{f}_{i}\right|+\left|{f{\prime\:}}_{i}\right|\:}$$

S^1^ ranges from 0 (asymmetry/dissimilarity) to 1 (perfect symmetry/similarity). When computed on distributions, it stands for the percentage of *K*-mer occurrences that are symmetrically distributed among complementary strands. Its complement to 1 (an asymmetry index) indeed corresponds to the weighted average of the absolute values of the skews of reverse-complementary bases or *K*-mers. Baisnée et al.^35^ also propose computing strand symmetry using Pearson’s linear correlation coefficient, *S*^*C*^, which ranges from − 1 to 1 and yieldsresults that are qualitatively similar to those obtained with S^1^.

### Measuring the Participation Ratio (PR)

In genomic sequences, it is widely recognized that over-represented *K*-mers, like stretches of As or Ts (poly(A) and poly(T) tracts), can skew the *S*^*C*^symmetry index^35^. Sequences with a more diverse *K*-mer distribution tend to produce higher *S*^*C*^ values. To prevent this bias, we propose another measure that is able to capture the main characteristics of the *K-*mer distribution: the participation ratio, or *PR*. Given an observed distribution of *K*-mers with relative frequencies *f*_i_, the PR for such distribution is calculated as:

3 $$\:PR=\frac{1}{{\sum\:}_{i=1}^{{4}^{K}}{f}_{i}^{2}}$$

The meaning of PR can be understood by considering two extreme situations: If all the *K*-mers appear with the same frequency, $\:{f}_{i}=1/{4}^{K}$ then PR =4^*K*^, i.e., all *K*-mers contribute (or *participate*) equally to the *K*-mer distribution. If only a single *K*-mer appears in the distribution, then $\:{f}_{i}=1$ for such *K*-mer and $\:{f}_{i}=0$ for the rest, and therefore PR = 1 since only one *K*-mer participates in the distribution. In general, PR indicates the number of *K*-mers participating effectively in the observed distribution. PR is commonly used in quantum solid-state physics to calculate the number of atoms where an electronic wave function is markedly different from 0 (see, for example^68^), .

## Electronic supplementary material

Below is the link to the electronic supplementary material.

Supplementary Material 1

Supplementary Material 2

## Data Availability

A list of the 4,336 fully sequenced genome sequences analyzed here, retrieved from the GISAID/Audacity database: GISAID EPI_SET_240824vr, available at https://doi.org/10.55876/gis8.240824vr.The following additional data and scripts are available at the open repository Zenodo (https://zenodo.org/records/14865335): -The rooted timetree in Newick format: timetree_1063.nwk-The Python script used to segment the SARs-CoV-2 coronavirus genome sequences and compute SCCs: SCC.zip, and its help file: SCC_readme.rtf -Python script for Stratified Sampling (https://zenodo.org/records/14870067).
