# Supplementary material for: Strong evidence for the evolution of decreasing compositional heterogeneity in SARS-CoV-2 genomes during the pandemic
Source: Sci Rep. 2025 Apr 10;15:12246. doi: 10.1038/s41598-025-95893-z (PMC11985940; doi:10.1038/s41598-025-95893-z)
Supplement: Supplementary file 1 — Supplementary Material 1 [file 41598_2025_95893_MOESM1_ESM.docx]

# Supplementary figures


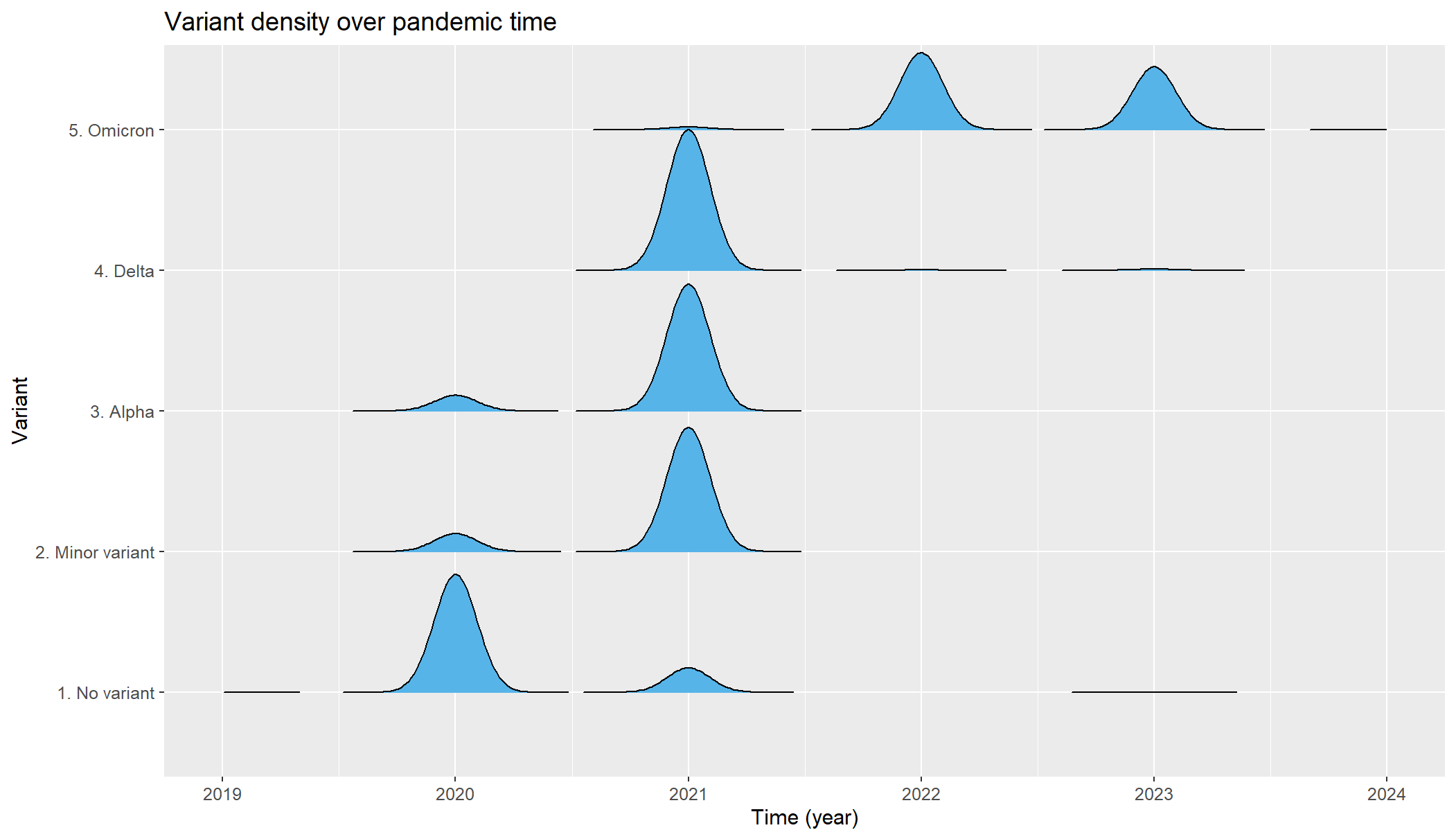


Supplementary Figure 1. Changing densities over time of main VOCs and other clades in the filtered sample with 4,336 completely sequenced genomes spanning from December 2019 to January 2024.


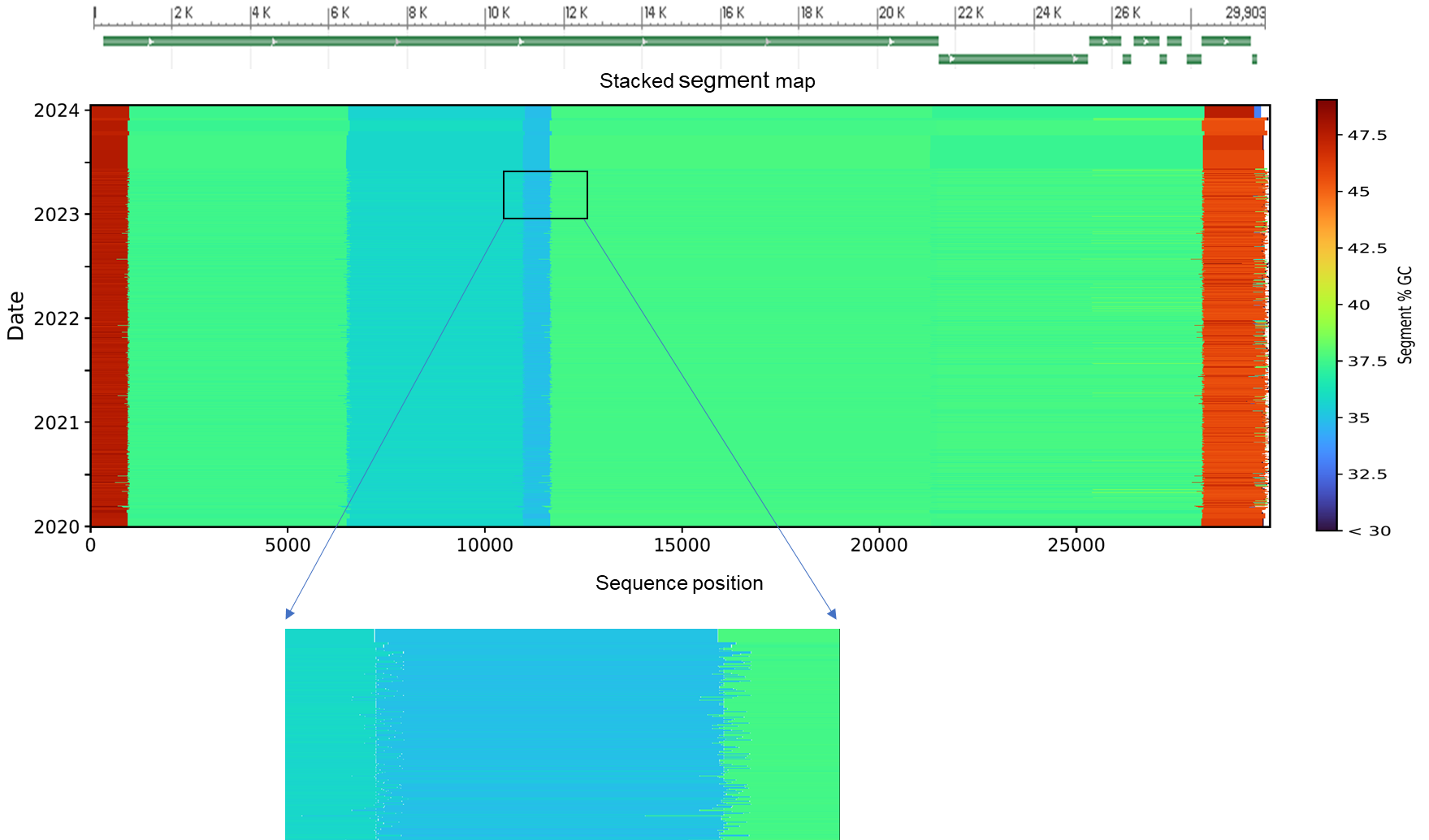


Supplementary Figure 2. Stacked graphical visualization map of the array of segments obtained from each genome, ordered by collection date. Each genome’s array of segments is depicted as a thin horizontal line, with colors standing for the %GC content of each segment (scale on the right). The zoomed region highlights the variation at segment boundaries across different genomes. A schematic gene map of the GenBank reference genome (MN908947.3, Wuhan-Hu-1) is displayed above. A more detailed view of the gene map is available at <https://www.ncbi.nlm.nih.gov/nuccore/MN908947.3?report=graph>.

| 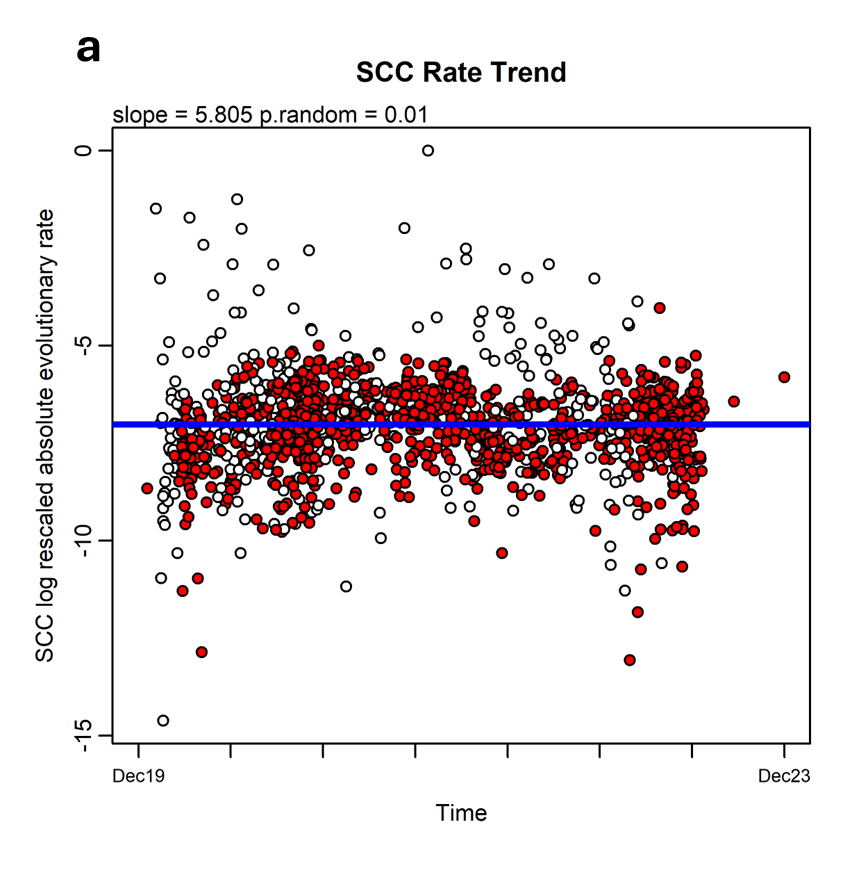 | 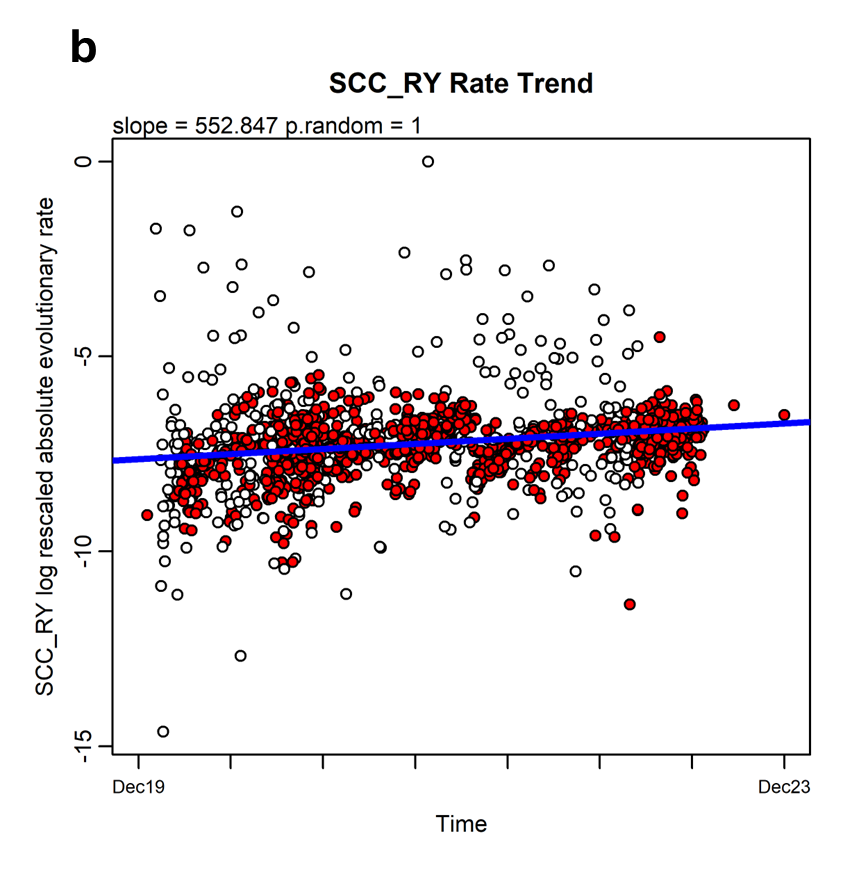 |
| --- | --- |

Supplementary Figure 3. Evolutionary rates of SCC (a) and SCC_RY (b) over pandemic time. See the caption of Figure 1 for annotations.
